# LG-UNet Based Segmentation and Survival Prediction of Nasopharyngeal Carcinoma Using Multimodal MRI Imaging

**DOI:** 10.3390/bioengineering12101051

**Published:** 2025-09-29

**Authors:** Yuhao Yang, Junhao Wen, Tianyi Wu, Jinrang Dong, Yunfei Xia, Yu Zhang

**Affiliations:** 1 College of Electronic Engineering (College of Artificial Intelligence), South China Agricultural University, Guangzhou 510642, China; 18938836048@139.com (Y.Y.); 943234708wjh@gmail.com (J.W.); wu1999jy@163.com (T.W.); 20243172011@stu.scau.edu.cn (J.D.); 2 State Key Laboratory of Oncology in South China, Guangdong Key Laboratory of Nasopharyngeal Carcinoma Diagnosis and Therapy, Guangdong Provincial Clinical Research Center for Cancer, Sun Yat-sen University Cancer Center, Guangzhou 510060, China; 3 State Key Laboratory of Oncology in South China, Department of Radiology, Sun Yat-sen University Cancer Center, Guangzhou 510060, China

**Keywords:** nasopharyngeal carcinoma, deep learning, image segmentation, metastasis prediction, LG-UNet

## Abstract

Image segmentation and survival prediction for nasopharyngeal carcinoma (NPC) are crucial for clinical diagnosis and treatment decisions. This study presents an improved 3D-UNet-based model for NPC GTV segmentation, referred to as LG-UNet. The encoder introduces deep strip convolution and channel attention mechanisms to enhance feature extraction while avoiding spatial feature loss and anisotropic constraints. The decoder incorporates Dynamic Large Convolutional Kernel (DLCK) and Global Feature Fusion (GFF) modules to capture multi-scale features and integrate global contextual information, enabling precise segmentation of the tumor GTV in NPC MRI images. Risk prediction is performed on the segmented multi-modal MRI images using the Lung-Net model, with output risk factors combined with clinical data in the Cox model to predict metastatic probabilities for NPC lesions. Experimental results on 442 NPC MRI scans from Sun Yat-sen University Cancer Center showed DSC of 0.8223, accuracy of 0.8235, recall of 0.8297, and HD95 of 1.6807 mm. Compared to the baseline model, the DSC improved by 7.73%, accuracy increased by 4.52%, and recall improved by 3.40%. The combined model’s risk prediction showed C-index values of 0.756, with a 5-year AUC value of 0.789. This model can serve as an auxiliary tool for clinical decision-making in NPC.

## 1. Introduction

Nasopharyngeal carcinoma (NPC) has a high incidence, particularly in southern China [1,2]. Distant metastasis in NPC poses a significant challenge in clinical treatment, making the effective identification and screening of patients at high risk of metastasis crucial for developing precise treatment plans and improving patient survival rates [1,2]. In recent years, deep learning techniques have been widely applied in NPC risk prediction. Neural network-based models enable automatic identification, precise segmentation, and feature extraction of NPC lesions from MRI images [3]. Building on this capability, these models facilitate the prediction of distant metastasis risk in NPC.

Currently, U-Net and its variants, based on convolutional neural networks (CNNs) [3,4,5,6,7,8,9], are widely adopted for medical image segmentation. CNNs effectively capture local image features through convolutional kernels of varying sizes [7]. However, their limited receptive fields, constrained by small kernel sizes, restrict their ability to model global patterns and contextual relationships. On the other hand, Vision Transformers (ViTs) [9,10,11], although originally developed for image classification, have recently been adapted for medical image segmentation to expand the receptive field; however, they exhibit weaker local feature extraction capabilities, often requiring additional strategies such as attention mechanisms to enhance segmentation performance [12,13,14,15,16].

To address these limitations, CNNs integrated with large convolutional kernels (LCKs) have been proposed. LCKs leverage surrounding tissue information to ensure a large receptive field while capturing large-scale features and contextual information that traditional CNNs struggle to obtain [17,18]. This makes LCK-based models particularly suitable for segmenting larger pathological structures [19]. In tasks involving audio, image, and other modalities [20,21], specifically adapted large-kernel [22,23,24] CNNs have demonstrated superior performance compared to ViTs. Furthermore, LCK modules with dynamic mechanisms enhance the modeling capability of tumor heterogeneity regions through adaptive weight adjustments.

However, the effectiveness of tumor image segmentation depends not only on the backbone network but also on the segmentation approach. Methods based on 2D [25,26], 3D [27,28,29], and 2.5D [30,31] segmentation all face challenges such as small spatial representation of lesions, background interference, and boundary ambiguity. The LVPA-UNet segmentation model [32], which integrates 2D and 3D parallel processing along with layer-channel attention mechanisms, addresses these issues to a significant extent. Nevertheless, its suboptimal skip connections and decoder design limit its ability to fully leverage contextual information.

To effectively capture large-scale features of nasopharyngeal carcinoma (NPC), this study proposes an improved LG-UNet segmentation model that expands the receptive field, leverages comprehensive contextual information, and addresses challenges such as ensuring adequate spatial representation of lesions, mitigating background interference, and resolving boundary ambiguity. The model integrates a dynamically cascaded large convolutional kernel (DLCK) mechanism [33] with a 2D and 3D parallel processing approach. The proposed LG-UNet model was applied to achieve precise segmentation of MRI images from 442 NPC cases obtained from the Sun Yat-sen University Cancer Center.

Although several models (e.g., DA-DSUNet, NPCNet, LVPA-UNet) [8,32,34] have improved NPC segmentation [35,36,37,38], and MRI-based approaches have been explored for survival prediction [39,40,41,42,43,44,45], most studies treat these tasks separately. Our work provides an integrated framework.

Building on the segmentation results, risk coefficients were extracted using the deep learning network Lung-Net [39]. These risk coefficients were then combined with clinical diagnostic information to construct a survival prediction model for NPC patients based on the Cox proportional hazards (CoxPH) model [40]. This approach provides a scientific foundation for developing individualized treatment strategies.

The main contributions of this study are as follows:

(a) Based on a 2D-3D hybrid network architecture, we propose the LG-UNet model for accurate gross tumor volume (GTV) segmentation of nasopharyngeal carcinoma (NPC) using multi-modality MRI images. This model assists clinicians in precise tumor staging.

(b) To complement the single T1-modality image dataset, T2 and T1-weighted images are introduced to enable multi-modality joint decision-making, thereby enriching the information available to the model.

(c) Leveraging the Lung-Net and Cox proportional hazards (CoxPH) models, transfer risk coefficients are extracted from high-dimensional features within the segmented regions to facilitate survival prediction.

## 2. Materials and Methods

### 2.1. Network Encoder

**Figure 1:**
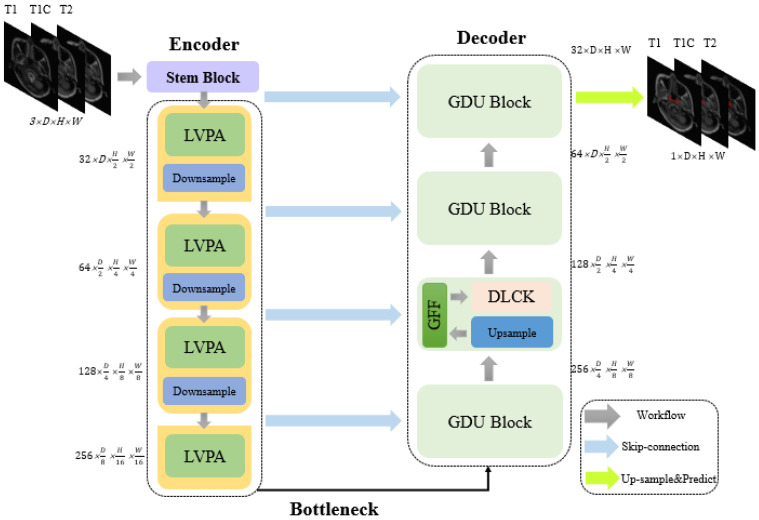
The proposed overall architecture of LG-UNet. Abbreviation: LVPA = Layer-Volume Parallel Attention.

The LG-UNet employs the encoder architecture described in reference [1], organized in a four-stage cascading manner. In Figure 1, these stages work together to gradually reduce the resolution of feature maps while extracting richer semantic features. This structured encoder cleverly integrates the Layer-Volume Parallel Attention(LVPA) module with the Overlapping Patch Embedding down-sampling operation, significantly improving the network’s performance in medical image segmentation tasks. The LVPA module, comprising 2D-MSCA, 3D-MSCA, and channel-wise attention modules, enables the simultaneous extraction of 2D slice and 3D volume features. The channel-wise attention module emphasizes tumor-related features, enhancing the network’s ability to identify lesion areas. Meanwhile, the Overlapping Patch Embedding down-sampling operation divides the image into multiple patches for processing using a sliding window approach. Furthermore, the Skip Connection strategy in the encoder not only preserves feature information at different scales but also ensures effective fusion of these features during the decoding stage, enabling comprehensive utilization of both detailed and contextual information. This encoder design allows the network to generate more discriminative feature maps, achieving finer tumor segmentation.

### 2.2. Network Decoder

The decoder of LG-UNet consists of four stages, each containing a group of GDU modules. These modules work synergistically to effectively extract multi-scale features and integrate global contextual information, providing rich feature representations for the decoder stage. The construction of feature dimensions in the decoder corresponds to that of the encoder and are designed to retain as much image feature information as possible, considering the relatively low D average layer depth in the dataset used in this study.

### 2.3. The Architecture of the GDU Module

**Figure 2:**
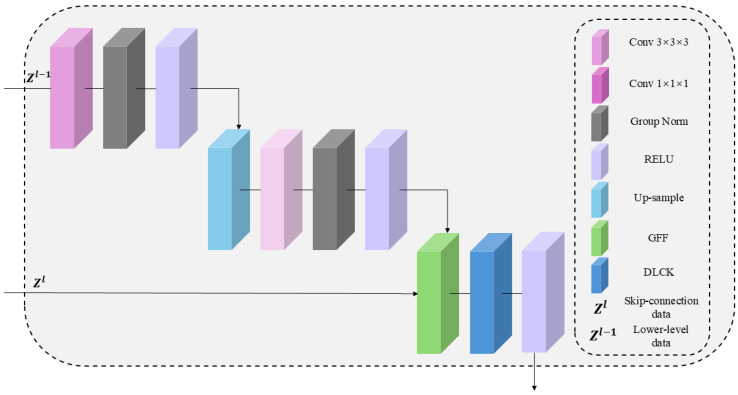
The architecture of the proposed GDU module in detail. Abbreviation: Conv = convolution; RELU = rectified linear unit; Group Norm = group normalization.

The specific architecture is shown in Figure 2. The GDU module is a key component of LG-UNet, composed of convolutional layers such as Conv3 and Conv1, Group-Norm batch normalization layer, RELU activation function, and the GFF and DLCK modules.

#### 2.3.1. The Overview of GFF Module

**Figure 3:**
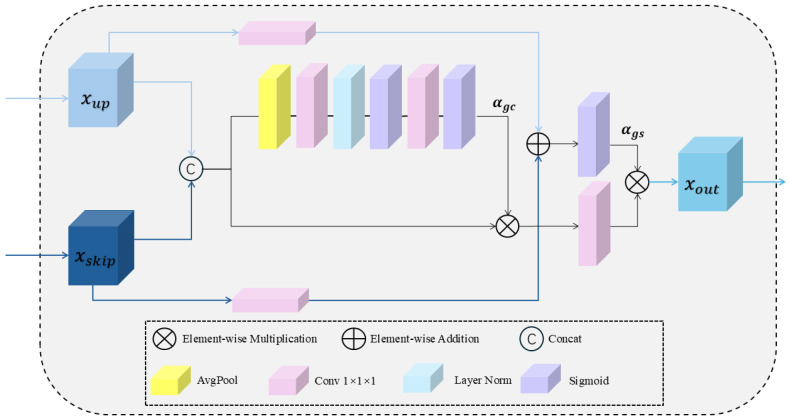
Detailed architecture of the Global Feature Fusion (GFF) module, illustrating the process of integrating multi-scale features through skip connections and feature upsampling techniques.

Based on the feature fusion module described in reference [2], the number of feature channels and convolution layers has been optimized, leading to the development of a Global Feature Fusion (GFF) module. In Figure 3, this module adaptively fuses multi-scale local features based on global information. In traditional U-Net upsampling, due to the limitations of the operation itself, feature maps often experience dilution of information and loss of details as the resolution increases. This leads to increased sparsity of feature maps and blurred details, which in turn affects the accuracy of segmentation results and the clarity of edges. Global information carries more complete contextual information, not only complementing fine details that may be lost during the upsampling process, such as texture and edge information, but also providing a deeper understanding of the overall structure of the image. Therefore, the incorporation of global information can effectively address the issue of feature information loss and detail blurring caused by upsampling.

Specifically, the GFF module receives feature maps $x_{up}$ and $x_{skip}$ from the skip connections and upsampling, respectively, for feature concatenation. The module then applies cascaded average pooling (AvgPool), two 1 × 1 × 1 convolution operations (Conv1), and two Sigmoid activations to first expand and then compress the feature channels, thereby extracting more detailed global channel descriptors. This enables the model to assign attention weights based on the overall performance of each channel across the entire spatial domain. This operation can be succinctly expressed as:

(1) $$\alpha_{gc}\;=\;\mathrm{Sigmoid}\;(\mathrm{Conv}1\;(\mathrm{Sigmoid}\;(\mathrm{Conv}1\;(\mathrm{AvgPool}\;([x_{up};\;x_{skip}])))))$$

Based on the obtained channel information weights, key features in the concatenated feature map are enhanced, while relatively less important features are suppressed. This helps the model to focus on the more relevant features for the task. Subsequently, a 1 × 1 × 1 convolution (Conv1) operation is applied again to extract more discriminative feature maps. This operation can be succinctly expressed as:

(2) $$x_{\alpha }=\;\mathrm{Con}v_{1}\;\left(\alpha_{gc}⊗[x_{up};x_{skip}\right])$$

At the same time as the above two steps, 1 × 1 × 1 convolution (Conv1) operations are applied to the feature maps $x_{up}$ and $x_{skip}$, followed by feature multiplication to obtain the global spatial location feature $\alpha_{gs}$. $\alpha_{gs}$ reflects the importance of each spatial position within the entire feature representation, including both $x_{up}$ and $x_{skip}$. This operation can be succinctly expressed as:

(3) $$\alpha_{gs}=\;\mathrm{Sigmoid}\;(\mathrm{Con}v_{1}\;(x_{up})\;⊕\;\mathrm{Con}v_{1\;}(x_{skip}))$$

(4) $$x_{out}=\alpha_{gs}\;⊗\;x_{\alpha }$$

#### 2.3.2. The Overview of DLCK Block

Traditional convolutional neural networks (CNNs) typically employ small convolutional kernels, such as 3 × 3 × 3 or 5 × 5 × 5, which limit the receptive field and hinder the ability to capture comprehensive contextual information. This study incorporates the Dynamic Large Convolutional Kernel (DLCK) block, as referenced in [2], introducing larger convolutional kernels, such as 5 × 5 × 5 and 7 × 7 × 7, to expand the receptive field. Since the upsampling operation reduces feature map resolution, applying the DLCK block before upsampling in the decoder helps the model better retain fine-grained details that would otherwise be lost. Unlike traditional CNNs that employ parallel multi-kernel structures, the DLCK block adopts a cascaded approach, progressively increasing kernel size and dilation rates. This cascaded design offers two distinct advantages: (a) recursively aggregating contextual information within the receptive field, continuously expanding the effective receptive field. (b) Assigning different weights to features based on their respective receptive field sizes, enabling deeper features from larger receptive fields to contribute more significantly to the final DLCK output.

**Figure 4:**
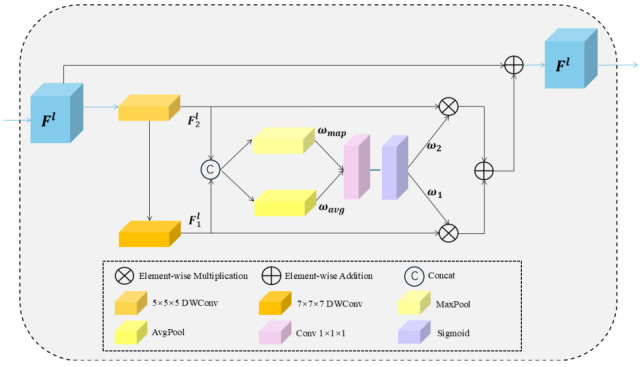
Detailed architecture of the Dynamic Large Conventional Kernel (DLCK) module, illustrating the process of enhancing model performance through dynamically adjusting large-sized convolution kernels and integrating multi-scale feature extraction techniques.

Figure 4 illustrates the overall structure of the DLCK block. Given an input feature map $F^{l}\in R^{C\times D\times H\times W}$ at layer l, a 5 × 5 × 5 depth-wise convolution with a dilation rate of 1, denoted as DWConv (5,1), is first applied, followed by a 7 × 7 × 7 depth-wise convolution with a dilation rate of 3, denoted as DWConv (7,3). This sequential operation assimilates local information while progressively expanding the effective receptive field. This process can be concisely expressed as:

(5) $$F_{1}^{\iota }=\;\mathrm{DWCon}{v\;}_{\left(5,1\right)}(F^{l})$$

(6) $$F_{2}^{\iota }=\mathrm{DWCon}{v\;}_{\left(7,3\right)}(F_{1}^{\iota })$$

The cascaded design enables the DLCK block to achieve an effective receptive field equivalent to a 23 × 23 × 23 convolution kernel, significantly reducing computational costs. Subsequently, $F_{1}^{\iota }$, $F_{2}^{\iota }$ are concatenated and processed separately using max pooling and average pooling to obtain dimension-reduced features. A 7 × 7 × 7 convolution layer, denoted as ${Conv}_{7}$, followed by a Sigmoid activation, is then applied to generate dynamic weights $\omega_{1},\omega_{2}$, which adaptively determine the relative importance assigned to different features.

(7) $$\omega_{avg}\;=Avgpool([F_{1}^{\iota };\;F_{2}^{\iota }])$$

(8) $$\omega_{map}\;=Maxpool([F_{1}^{\iota };\;F_{2}^{\iota }])$$

(9) $$[\omega_{1};\;\omega_{2}]=\mathrm{Sigmoid}\;(\mathrm{Con}v_{7}\;([\omega_{avg};\;\omega_{map}]))$$

The dynamic weights guide the calibration of features extracted from different convolution kernels. Finally, a residual connection is applied to ensure that no original information is lost and to mitigate the risk of overfitting.

(10) $$F^{l}\;=((\omega_{1}⊗F_{1}^{\iota })\;⊕\;(\omega_{2}⊗F_{2}^{\iota }))+F^{l}$$

The DLCK module is implemented by integrating the DLCK block into two convolutional layers with GELU activation and residual connections. It comprises feature extraction, global spatial relationship modeling, dynamic selection value generation, and feature calibration output. The operations performed by the DLCK module can be concisely expressed as:

(11) $$F^{l}=\;\mathrm{Con}v_{1}\;\;(F^{l-1})$$

(12) $$F^{l}=\mathrm{DLCK}\;(GELU\;(F^{l}))$$

(13) $${\hat{F}}^{\iota }\;=\mathrm{Con}v_{1}\;(F^{l})+F^{l-1}\;$$

LG-UNet is built upon a CNN architecture, incorporating a DLCK module and an MLP (Multilayer Perceptron) module following the global feature fusion operation in the encoder. This enables the network to capture multi-scale contextual information within the fused features, thereby enhancing feature representation.

### 2.4. Survival Prediction Task

LungNet is a deep learning model specifically designed for medical image segmentation tasks, particularly in lung imaging. In this study, the model was fine-tuned based on the task scenario, utilizing images segmented by LG-UNet. A multilayer perceptron (MLP), composed of a series of fully connected layers, batch normalization layers, ReLU activation functions, and dropout layers, was employed to extract and transform key features while effectively implementing regularization. Through the combined effect of these layers, the model precisely captures and identifies critical information from the high-dimensional features within the segmented regions that are decisive for metastasis risk assessment. Ultimately, the model outputs a quantitative risk score, which, when integrated with clinical information, facilitates further survival prediction.

The Cox proportional hazards (CoxPH) regression model is a commonly used semi-parametric model in survival analysis, designed to analyze the impact of covariates on survival time while accounting for censoring. It operates under assumptions such as proportional hazards, linearity, and independence. The regression coefficients are typically estimated using the maximum likelihood estimation (MLE) method. The fundamental form of the model is expressed as:

(14) $$h(t|X)=h_{0}(t)exp(\beta_{1}X_{1}+\beta_{2}X_{2}+\cdots +\beta_{p}X_{p})$$

After obtaining the risk scores from the model, the five-year ROC curve is plotted by computing the true positive rate (sensitivity) and false positive rate at different risk score thresholds. Additionally, the Youden index is calculated based on these values. By iterating through all possible thresholds, the threshold corresponding to the maximum Youden index is determined as the optimal threshold.

Based on the identified optimal threshold, patients are classified into high-risk and low-risk groups. The Kaplan–Meier method is then used to generate survival curves. The log-rank test is performed to assess the statistical significance of survival differences between the high-risk and low-risk groups, thereby evaluating the effectiveness of the risk score combined with clinical information in survival prediction.

## 3. Data

### 3.1. Image Segmentation Task Data

**Figure 5:**
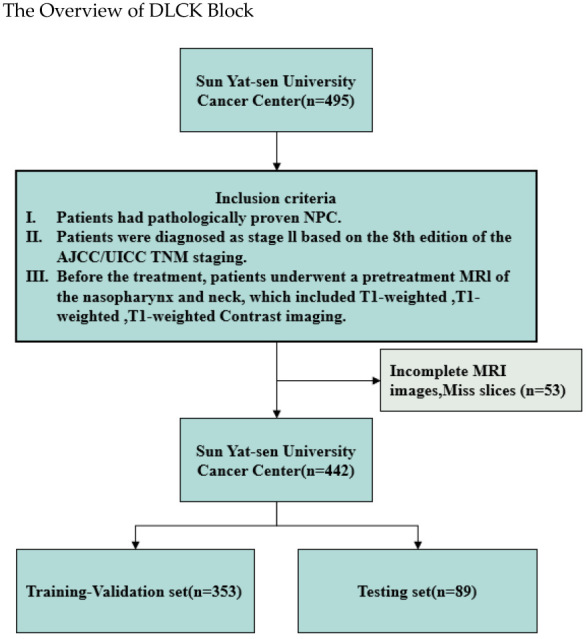
Flowchart of patient enrollment. NPC = nasopharyngeal carcinoma, AJCC/UICC = American Joint Committee on Cancer/Union for International Cancer.

This study retrospectively analyzed 495 stage II nasopharyngeal carcinoma (NPC) patients from Sun Yat-sen University Cancer Center, with data collected between May 2010 and July 2017 in Figure 5. Based on the inclusion and exclusion criteria and the selection process, a total of 442 NPC patients with corresponding diagnostic MRI scans (median age: 44 years; 310 males, 132 females) were included in this study. The dataset was randomly divided into training, testing, and validation sets in a 3:1:1 ratio. Each patient’s MRI data consisted of three modalities: axial T1-weighted (T1W), T2-weighted (T2W), and contrast-enhanced T1-weighted (T1W) images. Two experienced radiation oncologists manually delineated the gross tumor volume (GTV) on all MRI slices following institutional guidelines. Discrepancies were resolved by consensus with a senior oncologist, and the final contours served as the ground truth masks for training and evaluation.

Prior to the experiments, all three-modal MRI datasets underwent uniform preprocessing. In addition to pixel value normalization, data augmentation techniques, including random flipping and rotation, were applied to enhance the model’s generalization ability. Furthermore, to ensure consistent voxel spacing and image dimensions, the MRI images were resampled using trilinear interpolation, with voxel spacing set to 0.5 × 0.5 × 6 mm^3^ and dimensions adjusted to 32 × 256 × 256, which adequately covering all tumor lesions. For samples exceeding the specified dimensions, boundaries along the x, y, and z axes were cropped, ensuring that the cropping did not affect the lesion areas.

### 3.2. Survival Prediction Task Data

The clinical and treatment information collected in this study is shown in Table 1, including age, gender, T stage, N stage, lactate dehydrogenase (LDH), Epstein–Barr virus deoxyribonucleic acid (EBV DNA), and distant metastasis-free survival (DMFS), among others. For missing LDH and EBV DNA data, the missing values were imputed using the mean value. Regarding the follow-up plan, patients were followed up every three months during the first two years after admission, then every six months, and finally annually, in order to monitor the occurrence of distant metastasis. Distant metastasis-free survival (DMFS) was used as the primary clinical outcome.

#### Survival Prediction Data Preprocessing

To balance the number of positive and negative samples, a subset of 388 patients’ clinical information and corresponding deep learning image features was randomly selected from the original dataset for survival prediction. To address missing information in the clinical data, data cleaning was performed to remove obvious input errors and anomalous values, ensuring dataset accuracy and consistency. For missing numerical data, mean imputation was applied, filling missing values with the average of each feature to maintain the overall data distribution. Given the relatively small sample size, five-fold cross-validation was employed in survival prediction experiments to fully utilize the limited data and enhance the model’s reliability and generalization capability.

### 3.3. Performancce Metrics

#### 3.3.1. Segmentation Task Evaluation Metrics

The Dice Similarity Coefficient (DSC) is a region-based metric used to evaluate the overlap between the predicted values (*P*) and the ground truth values (*G*). The definition of DSC is as follows:

(15) $$DSC(P,G)=\frac{2\left|P\cap G\right|}{\left|P\right|+\left|G\right|}$$

DSC ranges from 0 to 1, with a higher DSC indicating better performance.

HD95 measures the boundary discrepancy between the prediction and the ground truth, where lower values indicate better segmentation performance. Let $Z_{pred}$ represent the boundary of the predicted mask, and $Z_{true}$ represent the boundary of the ground truth mask. The maximum HD is defined as:

(16) $$max\left\{h\left(Z_{pred},Z_{true}\right),h\left(Z_{true},Z_{pred}\right)\right\}$$

(17) $$h\left(Z_{pred},Z_{true}\right)=max_{a\in Z_{pred}}min_{b\in Z_{true}}\left|\left|a-b\right|\right|$$

(18) $$h\left(Z_{true},Z_{pred}\right)=max_{a\in Z_{true}}min_{b\in Z_{pred}}\left|\left|a-b\right|\right|$$

The 95% Hausdorff Distance (HD95) is adopted to reduce the impact of a small number of outliers.

#### 3.3.2. Survival Prediction Task Evaluation Metrics

The Time-Dependent Receiver Operating Characteristic (TD-ROC) curve is primarily constructed by calculating sensitivity and specificity at a specific time point t.

(19) $$Sensitivity\left(t\right)=\frac{TP\left(t\right)}{TP\left(t\right)+FN\left(t\right)}$$

(20) $$Specificity\left(t\right)=\frac{TN\left(t\right)}{TN\left(t\right)+FP\left(t\right)}$$

In this context, *TP*(*t*) represents the number of individuals correctly predicted as experiencing the event by time t or earlier (true positives), while *FN*(*t*) denotes the number of individuals for whom the event actually occurred but was not predicted within this timeframe (false negatives). Similarly, *TN*(*t*) indicates the number of individuals accurately predicted as not experiencing the event (true negatives), whereas *FP*(*t*) refers to the count of individuals incorrectly predicted to experience an event that did not occur (false positives).

The Area Under the Curve (AUC) serves as a comprehensive metric for evaluating the predictive accuracy of a model. At a specific time point *t*, the AUC can be expressed as:

(21) $$AUC\left(t\right)=\int_{0}^{1}Sensitivity\left(t|FP\;Rate\right)d\left(FP\;Rate\right)$$

where the False Positive Rate (FP Rate) is defined as 1−Specificity1−Specificity. The AUC ranges from 0.5, indicating no predictive ability, to 1.0, representing perfect predictive performance.

The Concordance Index (C-Index), quantifies the ability of a given model to correctly predict the order of survival times. The C-Index ranges from 0.5 to 1.0, where a value of 0.5 indicates purely random predictions, signifying that the model has no predictive power, and a value of 1.0 denotes perfect concordance between the model’s predictions and the observed outcomes. The formula for the C-Index is as follows:

(22) $$C-Index=\frac{\Sigma_{i,j}1\left(T_{i}>T_{j}\right)\cdot 1\left(r_{i}>r_{j}\right)\cdot \delta_{j}}{\Sigma_{i,j}\left(*T_{i}>T_{j}\right)\cdot \delta_{j}}$$

Here, $1\left(T_{i}>T_{j}\right)$ denotes a function that returns 1 if $\left(T_{i}>T_{j}\right)$,and 0 otherwise; $r_{i}$ represents the risk score of sample i; and $1\left(r_{i}>r_{j}\right)$ is a function that returns 1 when $r_{i}>r_{j}$, and 0 otherwise.

### 3.4. Implementation Details

**Figure A1:**
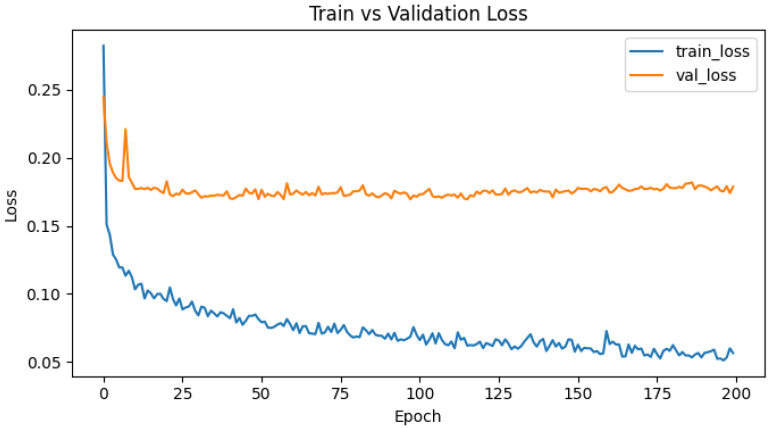
Training and validation loss curves of LG-UNet on the NPC MRI dataset.training/validation loss curves.

In this study, LG-UNet and subsequent survival prediction were implemented using PyTorch 1.11.0 + cu113 on a Windows Server equipped with a 12-core Intel Xeon CPU E5-2650 v4, 24 GB of RAM, and an NVIDIA GeForce RTX 3090. For the segmentation task, the Adam optimizer and a cosine scheduler were used with a batch size of 1. The initial learning rate was set to 1e-4, and training was performed for 300 epochs. To ensure reproducibility, we set the random seed to 1234, and experiments were run with Python 3.9.11, PyTorch 1.11.0 + cu113, CUDA 11.3, and cuDNN 8.2. The model contains 6.97 M parameters and 276.6 GFLOPs. Training and validation loss curves have also been included (see Figure A1 in the Appendix A).During the image testing process, the sliding window method from MONAI was applied, with a window size of 32 × 256 × 256 and an overlap rate of 50%. Validation was performed after each training epoch, and the model weights that achieved the highest validation performance were retained. In the segmentation experiment, the time for one training iteration was approximately 1200 s, and the time for one validation iteration was approximately 1700 s. All model parameters in both the image segmentation and survival prediction tasks remained consistent throughout this study.

## 4. Results

### 4.1. Analysis of Segmentation Experiment Results

#### 4.1.1. Comparison of Model Experiments

The proposed LG-UNet model was evaluated in comparison to a range of widely used medical image segmentation models, including 3D UNet [40], TransBTS [20], VT-UNet [19], UNETR [18], and LVPA-UNet [1]. As demonstrated in Table 2, LG-UNet consistently outperformed all competing models across multiple evaluation metrics, including the currently state-of-the-art LVPA-UNet.

Specifically, LG-UNet achieved an average Dice Similarity Coefficient (DSC) of 82.22%, surpassing LVPA-UNet’s 80.09% by nearly 2 percentage points. Additionally, LG-UNet exhibited superior performance in terms of HD95 (1.68 mm vs. 1.76 mm), precision (82.36% vs. 79.06%), and recall (82.97% vs. 82.29%) compared to LVPA-UNet. These results underscore LG-UNet’s enhanced accuracy in segmentation and classification tasks.

**Figure 6:**
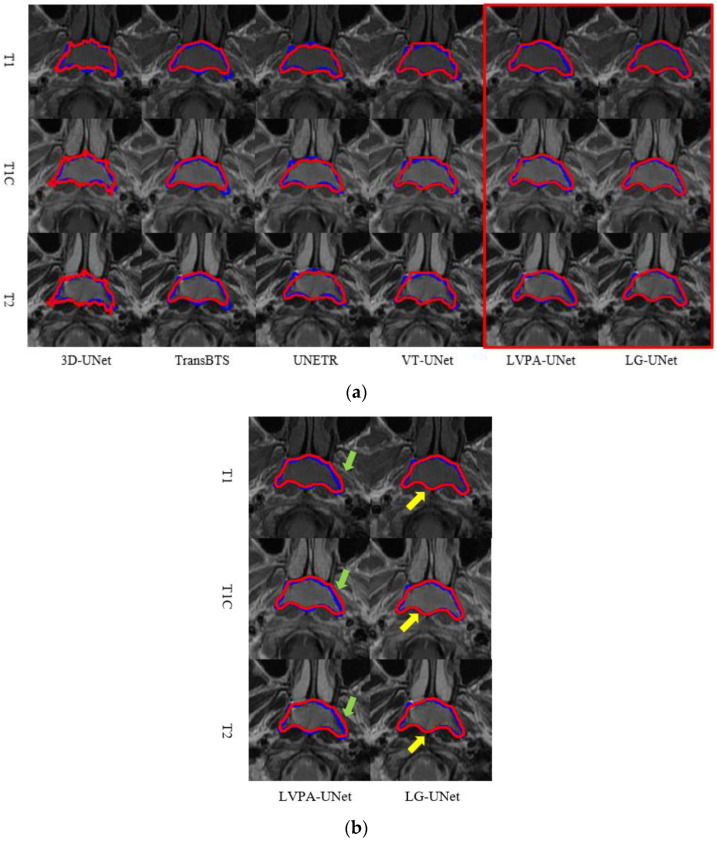

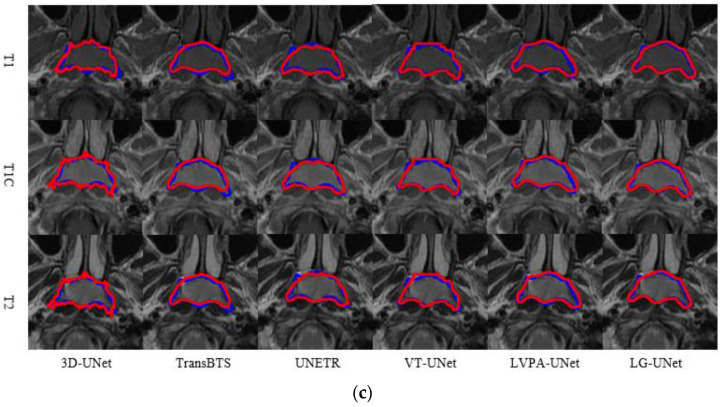
Real segmentation examples comparing LG-UNet with five other representative models. Sub-figure (**b**) provides an enlarged view of the region outlined in (**a**).

Figure 6a,b present real segmentation examples comparing LG-UNet with five other representative models. In these figures, the blue contours represent the ground truth annotations of the tumor GTV, while the red contours denote the segmentation predictions generated by the models. Each set of images, from top to bottom, displays T1, T1C, and T2 modality MRI slices of the same scan.

In Figure 6a, the segmentation results from 3D-UNet and TransBTS exhibit discontinuities and distortions in morphology. UNETR, VT-UNet, and LVPA-UNet also demonstrate varying degrees of inaccuracy, with noticeable discrepancies between their predictions and the ground truth. In Figure 6b, Areas of under- and over-segmentation by the baseline models are highlighted with green arrows. In contrast, yellow arrows point out the superior agreement of the LG-UNet boundaries with the ground truth. In contrast, Figure 6c shows that the predicted shapes from all six models generally align with the ground truth. However, 3D-UNet, TransBTS, UNETR, and VT-UNet display limited precision in capturing edge details. While LVPA-UNet performs better in edge detail preservation, its predictions extend beyond the ground truth boundaries. Notably, LG-UNet’s predictions in both slices closely match the ground truth, with superior edge detail representation. This observation highlights LG-UNet’s enhanced capability in processing edge details and deep features.

#### 4.1.2. Comparison of Module Ablation Experiments

To elucidate the specific contributions of each key component in LG-UNet, we conducted a series of ablation studies by progressively removing or modifying modules within the GDU (Gated Deformable Unit). The impact of these changes on the overall segmentation performance was quantitatively evaluated. As illustrated in Table 3, LVPA-UNet(U) [1] serves as the baseline model for the ablation experiments, where “U” denotes a cascaded decoder module containing only upsampling and its associated auxiliary components. “DU” represents a cascaded module incorporating the DLCK (Dynamic Large Convolutional Kernel), upsampling, and auxiliary components. “GU” refers to a cascaded module that includes the GDU, upsampling, and auxiliary components. Finally, “GDU” represents the complete GDU module.

**Figure 7:**
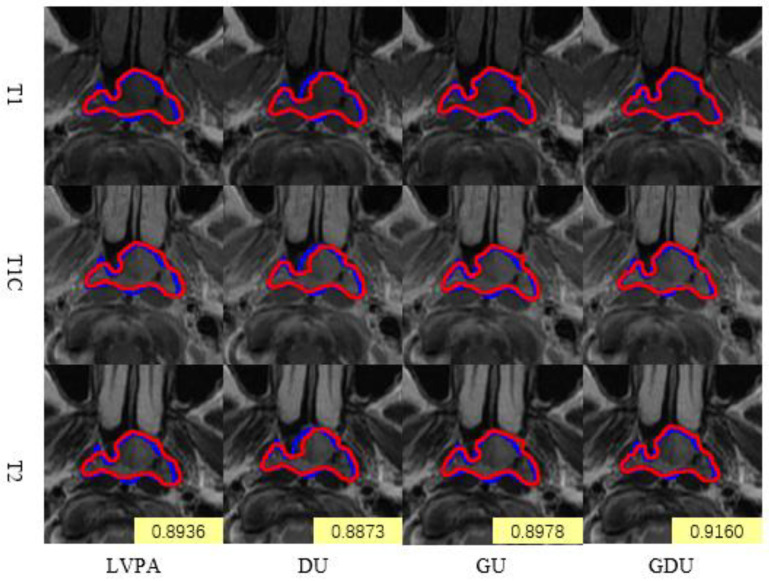
Real segmentation examples from the ablation experiments conducted on the individual modules of LG-UNet.

Figure 7 presents real segmentation examples from the ablation experiments conducted on the individual modules of LG-UNet. Each set of images, from top to bottom, displays T1, T1C, and T2 modality MRI slices of the same scan. In the segmentation experiments performed on the same case, all ablated models demonstrated relatively strong performance, achieving Dice scores of 0.8936, 0.8873, 0.8978, and 0.916, respectively. Compared to the baseline model, the DU model exhibited a slight performance decline, as the use of large convolutional kernels alone led to the omission of certain local details in the images. In contrast, the GU and GDU models showed improved accuracy in predicting the extent of the GTV lesion and better alignment with edge details compared to the baseline model. These results highlight the contributions of the GDU module to enhancing segmentation precision and detail preservation.

#### 4.1.3. Comparison of Multimodal and Single-Modality Inputs

The multimodal setting yielded higher Dice scores and lower HD95 compared to single-modality inputs, confirming that complementary anatomical and contrast information improves segmentation accuracy and clinical applicability, as detailed in Table 4.

### 4.2. Analysis of Survival Prediction Experiment Results

In this study, a number of widely used medical picture segmentation models based on the Lung-Net framework and images processed by LG-Unet, were subjected to risk coefficient extraction. The extracted features were combined with clinical information for further analysis. Due to the limited availability of clinical data, a 5-fold cross-validation approach was employed to train the Cox proportional hazards (CoxPH) model. In Table 5, the optimal Concordance Index (C-Index) achieved was 0.756, demonstrating the model’s predictive performance.

**Figure 8:**
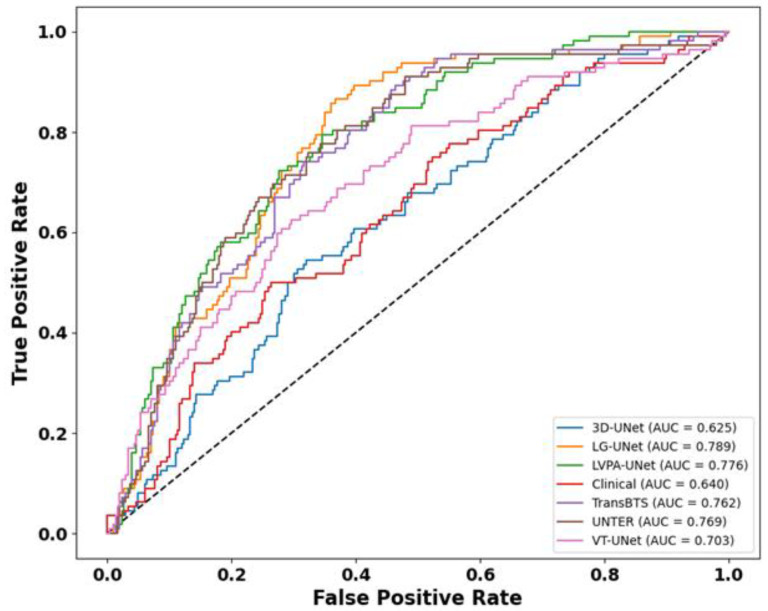
Comparison of TD-ROC curves for different models.

As illustrated in the TD-ROC curve, in Figure 8, LG-UNet achieves the highest predictive performance among all compared models, with its ROC trajectory consistently positioned closest to the upper-left corner and an AUC of 0.789. This substantially outperforms other architectures, underscoring its superior ability in time-dependent risk stratification and outcome discrimination. Additionally, LVPA-UNet and UNETR also demonstrate good performance, with AUC values ranging from 0.76 to 0.77. In contrast, 3D-UNet and the model based on clinical features exhibit poorer predictive capability, with AUC values of 0.625 and 0.640, respectively, and curves that are significantly offset from the top-left corner, demonstrating subpar predictive ability and a reduced capacity to differentiate between high-risk and low-risk populations.

**Figure 9:**
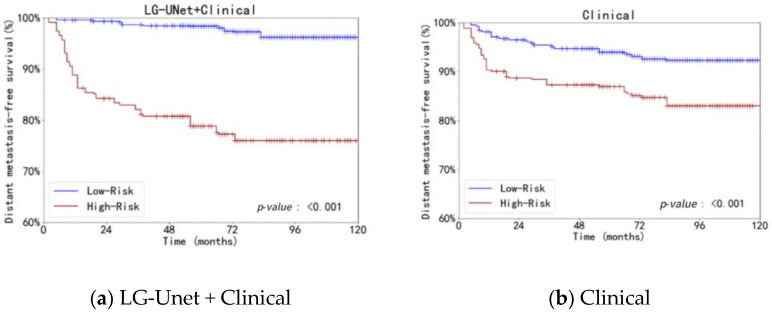
Comparison of Kaplan–Meier curves for different combination models.

The use of clinical data alone (Clinical) and clinical data combined with risk ratings obtained from LG-UNet (LG-UNet + Clinical) were compared in the Kaplan–Meier (KM) curve analysis. Both strategies showed a substantial difference in distant metastasis-free survival (DMFS) between the low-risk and high-risk groups (*p*-value: <0.001), as shown in Figure 9. The survival disparities between the risk groups were more clearly defined by the KM curve that included LG-UNet risk scores. This suggests that the risk scores produced by LG-UNet are an effective way to supplement clinical data and offer more discriminative information for patient survival prediction. In conclusion, LG-UNet demonstrates strong performance and potential clinical utility in survival prediction tasks.

## 5. Discussion

This study utilized a tri-modality MRI dataset comprising 442 cases of Stage II nasopharyngeal carcinoma from a single medical center. The gross tumor volume (GTV) annotations were meticulously delineated by two experienced radiation oncologists. Based on this dataset, we developed LG-UNet, a novel tumor segmentation model with multiple advantages. The Lung-Net prediction network was then used to obtain risk coefficients by utilizing the segmentation findings from LG-UNet. A survival prediction model was created by further integrating these risk coefficients with clinical data.

LG-UNet is an improved model based on the LCK-CNN framework, employing a 2D and 3D parallel processing approach for segmentation. In order to ensure precise spatial representation of lesions while reducing background interference and border ambiguity, it improves feature fusion operations and integrates a dynamic method for LCK (Large Convolutional Kernel) to enlarge the receptive field. LG-UNet achieves a Dice Similarity Coefficient (DSC) of 0.8223, precision of 0.8235, recall of 0.8297, and an HD95 of 1.6807 mm. Compared to the state-of-the-art LVPA-UNet model, LG-UNet demonstrates significant improvements, with a 7.73% increase in DSC, a 4.52% increase in precision, a 3.40% increase in recall, and a reduction of 0.0796 mm in HD95. Ablation experiments confirm that the model’s performance progressively improves with the integration of GFF (Global Feature Fusion) and DLCK (Dynamic Large Convolutional Kernel).

In comparison to other representative segmentation networks, LG-UNet exhibits superior performance in both segmentation results and accuracy, particularly in capturing edge details and precise delineation of tumor GTV. Deep learning models excel at extracting complex, disease-relevant features from medical images, which are often challenging to discern through traditional visual inspection. The survival prediction model built using the segmentation findings from LG-UNet has a five-year average AUC of 0.789 and a C-index of 0.756. LG-UNet has more accurate prediction performance when compared to models that use segmentation data from other networks. These experiments validate that the incorporation of deep learning features enhances the precision of metastasis prediction, offering potential benefits for the development of personalized treatment strategies.

This study has several limitations that should be acknowledged:

(a) Recently, the Mamba neural network architecture [41,42,43,44,45] has emerged as a promising approach due to its flexibility, enhanced feature representation capabilities, and computational efficiency. By incorporating hardware-aware parallelization design, this architecture achieves efficient modeling of long-range spatial dependencies while maintaining linear computational complexity, representing a significant breakthrough in architectural design. In the future, the integration of this architecture could serve as a potential solution for addressing challenges in medical image segmentation and research on distant metastasis.

(b) LG-UNet incorporates a dynamic large convolutional kernel mechanism and global information fusion at the decoder end, primarily optimizing skip connections and upsampling stages. Due to limited computational resources, the size and number of convolutional kernels in the large kernel mechanism were constrained. In the future, leveraging greater computational resources, dynamic large convolutional kernels or more sophisticated convolutional and fusion strategies could be introduced at the encoder end. Such improvements would enable more comprehensive utilization of image features and enhance the model’s ability to focus on critical features.

(c) The multi-modality MRI dataset used in this study is relatively limited in size, and a small portion of the corresponding clinical information is missing. These factors may constrain the accuracy of the statistical analysis for survival prediction. Additionally, the Lung-Net model employed in the prediction phase has limited capability in mapping deep features. In the future, the proposed methodology could be further validated using more extensive datasets and more advanced deep learning models.

(d) This study evaluated segmentation performance using DSC, HD95, precision, and recall. While additional metrics such as Surface Dice, ASSD, volumetric similarity, and boundary F-score, as well as stratified analyses by tumor size, contrast use, and imaging artifacts, could provide deeper insights, these were beyond the current scope and will be explored in future work.

## 6. Conclusions

An improved LG-UNet model is presented in this work for accurate GTV segmentation in nasopharyngeal carcinoma (NPC). A Global Feature Fusion (GFF) module optimizes skip connections and minimizes information loss during upsampling, while a Dynamic Large Convolution Kernel (DLCK) module broadens the receptive field and improves contextual feature representation. Furthermore, to handle spatial anisotropy and combine multimodal MRI data, a 2.5 D parallel processing approach is applied.

The LungNet model was modified for task-specific adjustments in terms of survival prediction. Using a multi-layer perceptron, high-dimensional characteristics were taken out of the LG-UNet segmentation findings and transformed into quantifiable risk coefficients. For the purpose of analyzing survival, these risk coefficients were input into a Cox proportional hazards model together with clinical factors. ROC curves and the Youden index were used to determine the ideal risk threshold, and patients were then split into high- and low-risk groups. Survival differences were evaluated using Log-Rank tests and Kaplan–Meier curves, confirming the model’s efficacy and potential for clinical use in predicting metastasis risk.

## Figures and Tables

**Table 1:** Statistical information of the dataset.

Characteristics	Dataset (*n* = 388)
**Age (years), median (IQR) ^6^**	44 (38.51)
**Sex, No (%**)	
Male	271 (69.9)
Female	117 (30.1)
**Histological type (WHO) ^6^, No (%)**	
WHO I	0
WHO II	20 (5.2)
WHO III	368 (94.8)
**T ^a^, No (%** **)**	
T1	197 (50.8)
T2	191 (49.2)
**N ^a^, No (%** **)**	
N0	48 (12.4)
N1	340 (87.6)
**LDH ^b^** **^6^, No (%)**	
Normal	369 (95.1)
Abnormal	19 (4.9)
Unknown	0
**EBV DNA ^c^** **^6^, No (%)**	
Undetectable	208 (53.6)
Detectable	161 (41.5)
Unknown	19 (4.9)
**DMFS** **^6^, No (%)**	
Metastasis-free	353 (91.0)
Metastasis	35 (9.0)

^a^ According to the 8th edition of the American Joint Committee on Cancer/Union for international Cancer Control cancer staging manual. ^b^ Abnormal, center 1: >245 U/L. ^c^ Detectable thresholds, center 1: <1000 copy/mL. ^d^ Data are represented as median (IQR). ^6^ Abbreviations: IQR: interquartile range; WHO, World Health Organization; LDH, lactate dehydrogenase; EBV DNA, Epstein–Barr virus deoxyribonucleic acid; DMFS, distant metastasis-free survival.

**Table 2:** Comparison of evaluation metrics of various typical models.

Models	DSC (%) ↑	HD95 (mm) ↓	Precision (%) ↑	Recall (%) ↑
3D UNet	76.31 ± 7.29	2.26 ± 1.41	76.88 ± 11.05	77.68 ± 10.74
TransBTS	77.85 ± 6.40	2.45 ± 1.92	78.45 ± 9.01	78.65 ± 10.10
UNETR	78.76 ± 6.87	2.19 ± 1.54	77.94 ± 8.84	79.71 ± 8.43
VT-UNet	78.67 ± 7.83	2.04 ± 2.13	78.36 ± 10.48	80.52 ± 10.49
LVPA-UNet	80.09 ± 7.25	1.76 ± 1.02	79.06 ± 10.46	82.29 ± 8.73
**LG-UNet**	**82.22 ± 6.47**	**1.68 ± 0.97**	**82.36 ± 7.96**	**82.97 ± 9.08**

**Table 3:** Comparison of evaluation metrics of various typical models.

Models	DSC (%) ↑	HD95 (mm) ↓	Precision (%) ↑	Recall (%) ↑
LVPA-UNet (U)	80.09 ± 7.25	1.76 ± 1.02	79.06 ± 10.46	82.29 ± 8.73
DU	79.31 ± 6.83	2.11 ± 2.55	79.02 ± 10.78	80.65 ± 10.51
GU	80.42 ± 6.89	1.75 ± 0.96	81.04 ± 9.81	81.01 ± 9.35
GDU	82.22 ± 6.47	1.68 ± 0.97	82.36 ± 7.96	82.97 ± 9.08

**Table 4:** Comparison of evaluation metrics of various typical models.

Input Channels	DSC (%) ↑	HD95 (mm) ↓	Precision (%) ↑	Recall (%) ↑
T1	77.18 ± 6.31	1.89 ± 1.02	79.05 ± 7.10	80.21 ± 6.55
T1C	80.05 ± 6.55	1.81 ± 0.96	80.10 ± 7.51	81.33 ± 7.48
T2	78.76 ± 6.71	1.93 ± 1.13	78.60 ± 6.68	79.94 ± 6.61
**T1 + T1C + T2**	**82.22 ± 6.47**	**1.68 ± 0.97**	**82.36 ± 7.96**	**82.97 ± 9.08**

**Table 5:** AUC and C-index values of different combined models.

Models	AUC (95%CI)	C-Index (95%CI)
CoxPH (3D UNet + Clinical)	0.625 (0.571–0.679)	0.584 (0.528–0.641)
CoxPH (TransBTS + Clinical)	0.762 (0.715–0.807)	0.726 (0.678–0.775)
CoxPH (UNETR + Clinical)	0.769 (0.725–0.812)	0.716 (0.667–0.784)
CoxPH (VT-UNet + Clinical)	0.703 (0.652–0.753)	0.694 (0.673–0.762)
CoxPH (LVPA-UNet + Clinical)	0.776 (0.728–0.820)	0.734 (0.687–0.781)
**CoxPH (LG-UNet** **+ Clinical)**	**0.789** **(0.746–0.829)**	**0.756** **(0.699–0.804)**
CoxPH (Clinical)	0.640 (0.589–0.694)	0.636 (0.577–0.684)

## Data Availability

No new data were created or analyzed in this study. Data sharing does not apply to this article.

## Abbreviations

The following abbreviations are used in this manuscript:

LVPA	Layer Volume Parallel Attention
CNN	Convolutional Neural Network
NPC	Nasopharyngeal Carcinoma
AJCC/UICC	American Joint Committee on Cancer/Union for International Cancer Control
MRI	Magnetic Resonance Imaging
DMFS	Distant Metastasis Free Survival
LDH	Lactate Dehydrogenase
EBV DNA	Epstein–Barr Virus Deoxyribonucleic Acid
DSC	Dice Similarity Coefficient
C-Index	Concordance Index
TD-ROC	Time-Dependent Receiver Operating Characteristic
DLCK	Dynamic Large Convolutional Kernel
GFF	Global Feature Fusion
GELU	Gaussian Error Linear Unit
DWConv	Depthwise Convolution
